# Supplementary material for: Homologies between SARS-CoV-2 and allergen proteins may direct T cell-mediated heterologous immune responses
Source: Sci Rep. 2021 Feb 26;11:4792. doi: 10.1038/s41598-021-84320-8 (PMC7910599; doi:10.1038/s41598-021-84320-8)
Supplement: Supplementary file 1 — Supplementary Information 1. [file 41598_2021_84320_MOESM1_ESM.pptx]

## Slide 1
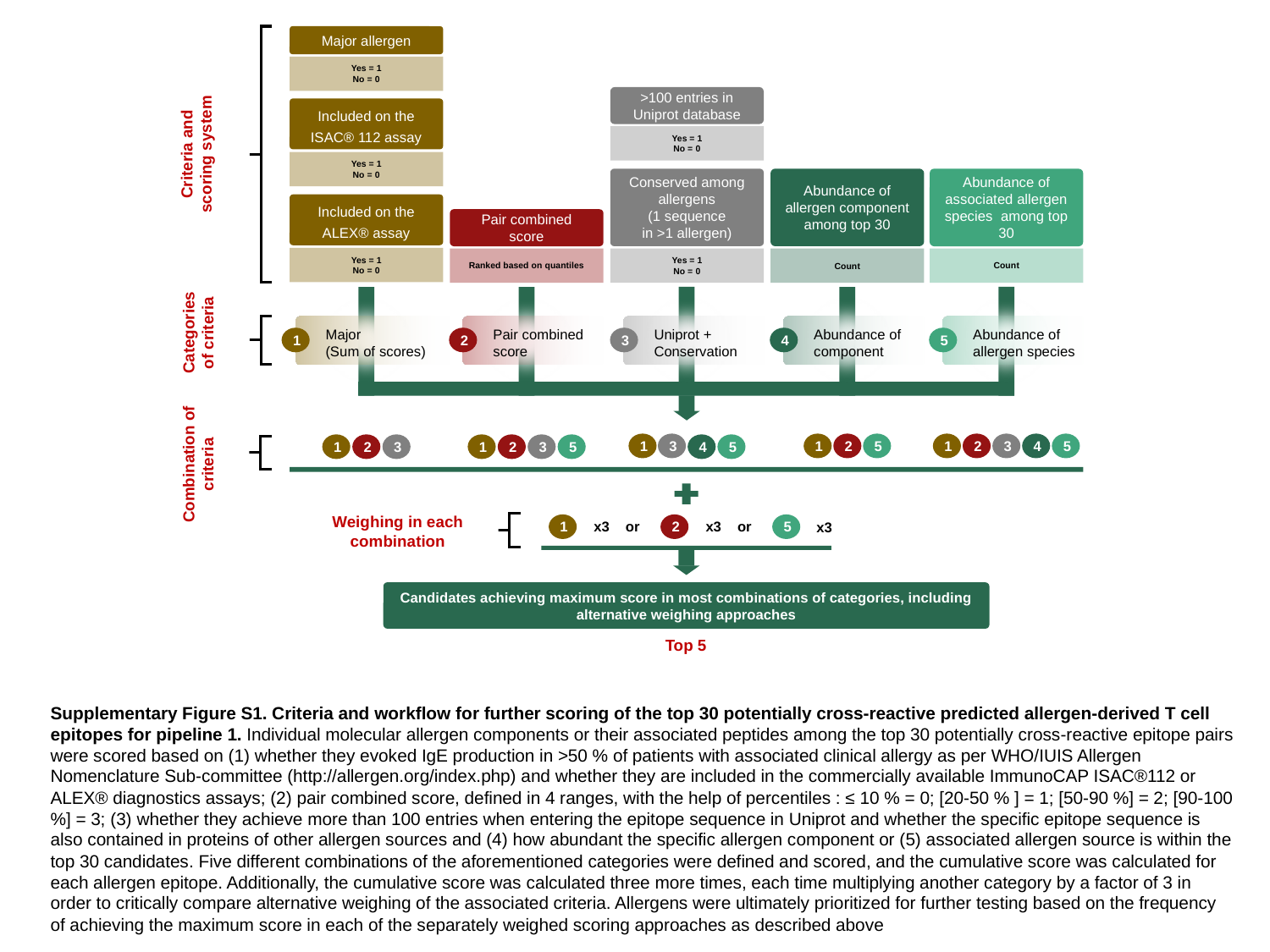

Major allergen
Yes = 1
No = 0
>100 entries in Uniprot database
Included on the ISAC® 112 assay
Yes = 1
No = 0
Criteria and scoring system
Yes = 1
No = 0
Conserved among allergens
(1 sequence
in >1 allergen)
Abundance of allergen component among top 30
Abundance of associated allergen species among top 30
Included on the ALEX® assay
Pair combined score
Yes = 1
No = 0
Ranked based on quantiles
Yes = 1
No = 0
Count
Count
Categories of criteria
Major
(Sum of scores)
1
Pair combined score
2
Uniprot + Conservation
3
Abundance of component
4
Abundance of allergen species
5
5
3
4
2
1
5
2
1
3
1
5
4
5
2
1
3
2
1
3
Combination of criteria
Weighing in each combination
1
2
5
x3 or
x3 or
x3
Candidates achieving maximum score in most combinations of categories, including alternative weighing approaches
Top 5
Supplementary Figure S1. Criteria and workflow for further scoring of the top 30 potentially cross-reactive predicted allergen-derived T cell epitopes for pipeline 1. Individual molecular allergen components or their associated peptides among the top 30 potentially cross-reactive epitope pairs were scored based on (1) whether they evoked IgE production in >50 % of patients with associated clinical allergy as per WHO/IUIS Allergen Nomenclature Sub-committee (http://allergen.org/index.php) and whether they are included in the commercially available ImmunoCAP ISAC®112 or ALEX® diagnostics assays; (2) pair combined score, defined in 4 ranges, with the help of percentiles : ≤ 10 % = 0; [20-50 % ] = 1; [50-90 %] = 2; [90-100 %] = 3; (3) whether they achieve more than 100 entries when entering the epitope sequence in Uniprot and whether the specific epitope sequence is also contained in proteins of other allergen sources and (4) how abundant the specific allergen component or (5) associated allergen source is within the top 30 candidates. Five different combinations of the aforementioned categories were defined and scored, and the cumulative score was calculated for each allergen epitope. Additionally, the cumulative score was calculated three more times, each time multiplying another category by a factor of 3 in order to critically compare alternative weighing of the associated criteria. Allergens were ultimately prioritized for further testing based on the frequency of achieving the maximum score in each of the separately weighed scoring approaches as described above
