# Supplementary material for: Homologies between SARS-CoV-2 and allergen proteins may direct T cell-mediated heterologous immune responses
Source: Sci Rep. 2021 Feb 26;11:4792. doi: 10.1038/s41598-021-84320-8 (PMC7910599; doi:10.1038/s41598-021-84320-8)
Supplement: Supplementary file 2 — Supplementary Information 2. [file 41598_2021_84320_MOESM2_ESM.docx]

**Supplementary Methods**

**Calculation of the pair combined score:**

The pair combined score takes the binding affinity of predicted viral and allergen epitopes to MHC molecules into consideration, as well as the score from the pairwise alignment and cross-entropy (cut-off 0,8): Pair combined score= 1/binding affinity (nM) (Virus) * 1/binding affinity (nM) (Allergen) * score PwA. Therefore, a higher score is associated with an increasing probability for MHC binding and a higher degree of similarity between the virus and allergen epitope.

**Scoring system:**

A scoring system was developed and five categories of criteria were formed, summing up the scores of individual criteria in one group (**Figure E1**). As a next step, five different combinations of the aforementioned categories were defined and the cumulative score was calculated for each allergen epitope. Additionally, the cumulative score was calculated three more times, each time multiplying another category by a factor of 3 in order to critically compare alternative weighing of the associated criteria. The new Top 5 allergens and associated virus epitopes were subsequently ranked based on the frequency of achieving the maximum score in each of the separately weighed scoring approaches as described above.
