# Supplementary material for: Homologies between SARS-CoV-2 and allergen proteins may direct T cell-mediated heterologous immune responses
Source: Sci Rep. 2021 Feb 26;11:4792. doi: 10.1038/s41598-021-84320-8 (PMC7910599; doi:10.1038/s41598-021-84320-8)
Supplement: Supplementary file 5 — Supplementary Information 5. [file 41598_2021_84320_MOESM5_ESM.docx]

**Supplementary Table S3: The most prevalent human HLA alleles, which were targeted for the in silico epitope prediction in pipeline 1**

| Human MHC Class I^1^ | Human MHC Class II^2^ |
| --- | --- |
| HLA-A*01:01 | DRB1*01:01 |
| HLA-A*02:01 | DRB1*03:01 |
| HLA-A*11:01 | DRB1*04:01 |
| HLA-A*24:02 |  |
| HLA-B*07:02 |  |
| HLA-B*40:02 |  |

1=epitopes with a length of 9 or 10 amino acids

2=epitopes with a length of 15 amino acids
